# Psychometric properties and measurement invariance of the short form of grit scale in Korean adolescents

**DOI:** 10.1371/journal.pone.0296795

**Published:** 2024-01-19

**Authors:** Sung-Woo Bae, Ji Geun Kim, Byung-Sun Park, Keungeun Lee, Jungkyu Park

**Affiliations:** 1 Division of Social Welfare, Kyungpook National University, Daegu, Republic of Korea; 2 Department of Psychology, Kyungpook National University, Daegu, Republic of Korea; 3 Department of Social Welfare, Gangneung-Wonju National University, Wonju-si, Gangwon State, Republic of Korea; University of Ha’il, SAUDI ARABIA

## Abstract

This study aimed to identify the factor structure of the Korean version of the Short Grit Scale (Grit-S) and examine its cross-sectional and longitudinal measurement invariance (MI). Data from the Korean Children and Youth Panel Survey 2018 were analyzed, which included two cohorts, comprising 2,327 and 2,325 fourth-year elementary and first-year middle school students, respectively. It was found that the two-factor model fit the data well for the elementary and middle school samples. The results of the cross-sectional MI tests across genders indicated that the full threshold and loading invariance were also supported for the elementary school sample, and the partial threshold and loading invariance were supported for the middle school sample. The analyses of the longitudinal MI revealed that the partial threshold and loading invariance were supported for both samples. The reliability analysis revealed satisfactory McDonald’s Omega values for both samples at each time point and moderate stability coefficients over time. Based on these findings, it was concluded that the Korean version of the Grit-S demonstrated satisfactory psychometric properties and exhibited MI across gender and time in Korean adolescents.

## Introduction

Grit is a psychological concept proposed by Duckworth et al. [1], which they defined as a personal, noncognitive attribute that reflects a person’s passion and perseverance for achieving long-term goals. Grit has two dimensions: consistency of interests (CI), which refers to the extent to which people can maintain a focused passion for a specific interest or goal over a long time, and perseverance of effort (PE), which refers to people’s inclination to uphold commitment and sustain exertion when confronted with challenges and adversity. Previous research has demonstrated that grit could be a measure for assessing positive educational, professional, and personal outcomes [2–4], and has been positively associated with school-related performances such as academic achievement, academic engagement, and school motivation [1, 5, 6]. People with high grit exhibit greater work retention and engagement and have fewer career changes (e.g.: [1, 6]). Grit also has associations with positive personal outcomes, such as psychological well-being, good mental health, and finding meaning in life [7–9].

To appropriately measure grit, Duckworth et al. [1] initially developed a 12-item Grit Scale, after which Duckworth and Quinn [10] improved the overall psychometric properties of the scale and validated an 8-item Short Grit Scale (Grit-S). Confirmatory factor analyses showed that the Grit-S fit data better than the Grit-O, and the predictive validity had not decreased even though there were fewer items, that is, the Grit-S was a more efficient grit measure. Consequently, the Grit-S was translated into many languages, including Korean [11], Japanese [12], Turkish [13, 14], German [15], Polish [16], Italian [17], Spanish [18, 19], Czech [20, 21], Chinese [22–24], Arabic [25], and Portuguese [26].

Although Grit-S has been the most frequently used grit measure, there have been concerns that the factor structure of Grit-S is inconsistent for different cultural groups [27]. The original factor structure of Grit-S, based on confirmatory factor analysis was a higher-order structure with two first-order factors, PE and CI [10]; however, factorial structural differences were found in collectivistic cultures. For example, the hierarchical Grit-S structure was not replicated in Filipino samples [28], and two items did not load significantly on the CI factor in Turkish samples [13]. Disabato et al. [27] analyzed the grit factor structure using culturally diverse samples from 109 countries and found that both CI and PE had low correlations in collectivistic cultures, and the CI and PE factor loadings on the general grit factor had variability even in collectivistic cultures. Those findings suggested that the Western individualistic concept of grit may be perceived differently in collectivistic social contexts [3, 29].

In addition to cultural differences, the grit factor structure may vary in different age groups. For example, Muenks et al. [30] compared the model fit indices for several educational levels and found that the bifactor model had a better fit for college students and that the two-correlated factor model was more appropriate for high school students. These findings suggested that people at different developmental stages may perceive grit differently. Unfortunately, most previous research has focused on validating the psychometric properties of grit using a wide range of samples, such as high school students [22, 31], college students [12, 13, 17, 18, 21, 26], and adult populations [16, 19, 24]. However, as the Grit-S factor structure in early adolescence has not yet been explored, the first objective of this study was to explore the grit factor structure in elementary and middle school students and the second objective was to investigate the Grit-S factor structure using samples from South Korea, a collectivist culture. Therefore, the findings of this study could shed light on the generalizability of the Western individualistic grit concept to a collectivist society.

A third objective was to assess the cross-sectional and longitudinal measurement invariance (MI) of Grit-S. Previous cross-sectional MI of Grit-S studies that have examined the associations between gender and grit have had mixed findings. For example, some studies have found that females have higher levels of grit than men [6, 10, 32, 33], while others found no gender differences [1, 26, 34]. These contradictory findings suggested that more research was needed to better understand the relationship between grit and gender and that establishing cross-sectional measurement invariance (CMI) was essential for accurately interpreting any observed mean differences between gender. In addition, several previous studies that examined MI for Grit-S across genders reported varying results across different age groups. For example, scalar invariance was found in middle school students [35], partial strict invariance in high school students [31], strict invariance in college students [26], and partial scalar or strict invariance in adult populations [16, 24]. However, due to the lack of gender-specific MI research with early adolescent samples, it remains unclear whether the Grit-S measures the same constructs in girls and boys in this age group. Therefore, this study also examined the MI of the Grit-S across genders in a sample of elementary and middle school students.

Given the widespread research interest in understanding the growth and changes in grit over time [36–39], it is also important to establish longitudinal measurement invariance (LMI) of the Grit-S. If there is evidence that shows that Grit-S measures the same construct with consistent structural validity across several time points, then observed changes in grit over time could be interpreted as genuine changes rather than changes in the construct’s structure or measurement. However, there is little research examining the LMI on Grit-S. To the best of our knowledge, only two cross-cultural studies have been conducted [23, 40], which makes it difficult to generalize. Furthermore, as these studies utilized analytical methods that did not account for the ordinal nature of Grit-S, this study examined the LMI of Grit-S by conducting a multigroup confirmatory factor analysis (CFA) using the unweighted least squares (UWLS) method following the guideline by Wu & Estabrook [41] to account for the ordered categorical nature of the Grit-S measurement.

As further research concerning the psychometric properties and measurement invariance of the Grit-S in early adolescents and collectivistic cultures is warranted, this study aimed to (a) explore the factor structure of the Grit-S in Korean elementary and middle school students, (b) assess the CMI to determine whether the Grit-S measures the same attribute across genders, and (c) examine the LMI at three time points over a one-year interval to assess the adequacy of the Grit-S for longitudinal comparisons.

## Materials and methods

### Participants

This study was conducted using data from the Korean Children and Youth Panel Survey 2018 (KCYPS 2018), which is a nationally representative longitudinal dataset collected by the National Youth Policy Institute using a stratified sampling approach [42]. The participant data were extracted using multistage cluster sampling. In total, 16 administrative districts were stratified, and schools were randomly selected from each district using proportionate probability sampling based on the population rate. Finally, one class from each school was randomly selected, and the data were collected annually from face-to-face interviews. The KCYPS 2018 consisted of two cohorts that included fourth-year elementary school students and first-year middle school students.

This study was approved by the Institutional Review Board of Kyungpook National University (IRB No. 2023–0170) and an informed consent was waived because the data were obtained from a public data depository which is freely available online. S1 Table presents the demographic characteristics of the sample participants.

The study examined the baseline data for both cohorts for CMI based on gender and separately analyzed the data for LMI from the baseline (2018) to the third survey year (2020) for each cohort. A total of 2,607 participants responded at baseline in 2018 for the elementary school sample; however, 170 (6.5%) and 110 (4.2%) participants dropped out in 2019 and 2020, making respective totals of 2,437 and 2,327 responses. Complete responses were received from 2,590 participants for the middle school sample in 2018; however, 152 (5.9%) and 113 (4.4%) participants dropped out in 2019 and 2020, making respective totals of 2,437 and 2,325 responses.

### Measures

The Grit-S [10] is a short version of the full Grit-O developed by Duckworth et al. [1]. The Grit-S evaluates trait-level perseverance and passion for long-term goals using two factors, CI and PE, each of which comprises four items. In the Korean version, each item of the self-reported Grit-S is rated using a four-point Likert-type scale ranging from 1 = *not at all like me* to 4 = *very much like me*.

Kim and Hwang [11] reported on the measurement translation procedures and measurement properties, including the reliability and validity, of the Korean Grit-S version. The authors translated and back-translated the scale and made the necessary corrections and revisions to derive the final Korean version, the reliability of which was assessed using Cronbach’s α (.714). The scale was also found to have concurrent validity, which confirmed its positive and significant correlations with academic motivation (*r* = .51) and self-control (*r* = .61).

### Data analysis

This study had four main objectives: (a) to explore the factor structure of the Grit-S using Korean elementary and middle school samples, (b) to evaluate the CMI of the Grit-S across genders, (c) to examine the LMI of the Grit-S over time, and (d) to investigate the reliability of the Grit-S scale. To achieve the first objective, exploratory factor analysis (EFA) was first conducted to explore the Grit-S factor structure without assuming any prior factor structure knowledge. Then CFA was conducted to determine whether the solution obtained from the EFA was retained. Due to the ordinal nature of the items, the EFA and CFA were conducted using the UWLS estimator with a polychoric correlation matrix.

To ensure the robustness of the findings, the elementary and middle school samples were randomly divided into two subsamples at time 1 (elementary school sample 1: *N* = 1,163; sample 2: *N* = 1,164; middle school sample 1: *N* = 1,162; sample 2: *N* = 1,163), after which the EFA and CFA were conducted on each subsample. Due to the inter-factor correlations, geomin rotation was used for the EFA to facilitate interpretability of solutions. Furthermore, parallel analysis was employed to determine the optimal number of factors to be extracted, which has been recognized as a suitable method for evaluating dimensionality in ordinal data with polychoric correlations [43].

A graphical technique called exploratory graph analysis (EGA) was adopted to validate the Grit-S factor structure’s EFA [44]. EGA is a network-based method to determine the dimensional structure of given items. Specifically, the Gaussian graphical model with polychoric correlations was adopted for the given items to establish a network, which was estimated using graphical least absolute shrinkage (GLASSO) with an extended Bayesian information criteria model selection operator owing to its capability to handle ordinal data [45]. The Louvain algorithm was applied to determine the optimal dimensions of the eight items, which provided information on which items belonged to each dimension [46]. The derived dimensions enabled a comparison of the EFA solution. A bootstrapping procedure was employed to assess the items’ stability and dimensional stability. Specifically, to assess whether the solution previously obtained was maintained, the item community assignments were estimated using 1,000 bootstrapped samples to determine which item belonged to each item community and the number of dimensions. The procedures were performed using the *EGAnet* package and the network visualization *qgraph* package [47].

Several fit indices were employed for the CFA to compare the goodness-of-fit of the proposed model, including the comparative fit index (CFI), the root mean square error of approximation (RMSEA), and the standardized root mean square residual (SRMR). The incremental fit measures, including a CFI greater than 0.95, were considered a good fit, with absolute fit measures, such as the RMSEA and SRMR, close to 0.06 indicating a reasonable fit [48].

Recent methodological studies have found that the fit index cutoff proposed by Hu and Bentler [48] could not be universally applied as a strict rule [49, 50], particularly when the UWLS or diagonally weighted least squares estimator are utilized to analyze ordered categorical data. In such cases, the conventional cutoff values exhibited worse performances in distinguishing misspecified models that fit poorly and correctly specified models that fit well, which led to models with significant misfits being considered acceptable [51–53]. To overcome such problematic performances of cutoffs with the categorical fit indices, the evaluation of the CFA models was further supplemented by generating the cutoffs with a dynamic fit index (DFI), which considered the specific characteristics of the models being fitted to derive the appropriate cutoff values [54]. R package *dynamic* [55] was used to customize the cutoff values that accommodated the ordered or categorical responses.

Subsequently, a multigroup CFA using the UWLS estimator was conducted to examine the MI of the Grit-S across genders at the baseline following the guidelines by Wu & Estabrook [41]. Specifically, a series of nested models were sequentially compared by constraining the model parameters to be equal across genders at each invariance test. The configural model was first estimated by freely estimating all parameters, followed by the model with equality constraints on the thresholds across gender, suggesting equivalent proportions for each response category. Subsequently, the model was estimated with equal thresholds and loadings, indicating similar measure for the constructs across gender. This approach was chosen over the conventional approach (e.g.: [56]) because the conventional approach, for which a baseline model is first established and then the constraints on loadings and thresholds are sequentially imposed, relies on how the baseline threshold structure is identified in relation to the latent response variable scales, which can potentially lead to misleading conclusions in MI tests.

Similarly, the LMI was then evaluated over time by equally constraining the model parameters across times in a series of nested models; configural, thresholds, and both threshold and loading invariance models. The MI was considered to be achieved if no significant differences were found in the fit indices between the more constrained and less constrained models. To determine the significance in the model fit changes, the Satorra–Bentler scaled chi-square difference test was utilized, which provided an appropriate evaluation for the differences between the nested models with ordered categorical indicators [57]. This approach computed the scaled chi-square statistics by dividing the standard chi-square statistics using scaling factors, which resulted in a better approximation of the chi-square distribution. A significant chi-square difference (Δχ^2^) indicated that the more constrained model had a poorer fit than the less constrained model.

In addition to the scaled chi-square difference test, a difference test based on the RMSEA value, referred to as RMSEA_D_, was also implemented [58] to assess the significance of the model fit changes. As the interpretation of this index was consistent with the conventional RMSEA value, a value more than .08 indicated that the more constrained model had a worse fit than the less constrained model due to the introduced constraints within the model. The *lavaan* [59] and *semTools* [60] packages in R were utilized to implement these procedures.

The Grit-S reliability was examined using internal consistency measures and stability coefficients. The internal consistency at each time point was evaluated based on McDonald’s ω, and the stability coefficient, which represented the correlation between the factors at two time points, was evaluated using a threshold and loading invariance model to assess the relative stability of the Grit-S. The analyses were performed using Mplus 8.4 [61], and the EGA was conducted using R 4.1.1 [62].

## Results

### Factor analysis and network estimation

Table 1 shows the descriptive statistics; means, standard deviations, skewness, and kurtosis; for the Grit-S at the three time points.

**Table 1:** Descriptive statistics of the Grit-S.

Factor and Item			Consistency of interest				Perseverance of effort			
			Item	Item	Item	Item	Item	Item	Item	Item
			1	3	5	6	2	4	7	8
**Elementary**	**Time 1**	**Mean**	2.75	2.90	2.69	2.93	2.88	2.96	2.88	2.78
		**Std. D**	0.85	0.83	0.82	0.85	0.81	0.76	0.81	0.76
		**Skewness**	-0.08	-0.20	-0.12	-0.30	-0.54	-0.42	-0.19	-0.18
		**Kurtosis**	-0.74	-0.78	-0.53	-0.72	-0.03	-0.10	-0.69	-0.35
	**Time 2**	**Mean**	2.65	2.74	2.59	2.76	2.83	2.85	2.76	2.69
		**Std. D**	0.77	0.78	0.74	0.77	0.73	0.73	0.76	0.71
		**Skewness**	0.10	-0.07	0.00	-0.09	-0.44	-0.30	-0.08	-0.13
		**Kurtosis**	-0.52	-0.49	-0.32	-0.46	0.19	-0.08	-0.47	-0.20
	**Time 3**	**Mean**	2.55	2.58	2.51	2.63	2.75	2.80	2.73	2.61
		**Std. D**	0.74	0.77	0.74	0.79	0.73	0.69	0.71	0.72
		**Skewness**	0.29	0.03	0.06	-0.05	-0.35	-0.16	0.00	-0.13
		**Kurtosis**	-0.40	-0.42	-0.30	-0.46	0.04	-0.14	-0.37	-0.21
**Middle**	**Time 1**	**Mean**	2.54	2.61	2.60	2.72	2.72	2.79	2.73	2.56
		**Std. D**	0.77	0.76	0.74	0.77	0.74	0.74	0.77	0.79
		**Skewness**	0.17	0.11	0.02	-0.10	-0.26	-0.14	0.01	0.01
		**Kurtosis**	-0.42	-0.46	-0.35	-0.41	-0.13	-0.32	-0.55	-0.44
	**Time 2**	**Mean**	2.55	2.56	2.57	2.68	2.69	2.73	2.70	2.56
		**Std. D**	0.73	0.75	0.72	0.72	0.71	0.74	0.71	0.72
		**Skewness**	0.17	0.24	0.01	-0.01	-0.25	-0.20	0.01	-0.10
		**Kurtosis**	-0.34	-0.41	-0.28	-0.33	-0.05	-0.22	-0.34	-0.24
	**Time 3**	**Mean**	2.48	2.50	2.55	2.60	2.63	2.72	2.62	2.47
		**Std. D**	0.72	0.76	0.73	0.76	0.74	0.73	0.71	0.74
		**Skewness**	0.26	0.23	0.04	0.04	-0.34	-0.17	-0.03	0.03
		**Kurtosis**	-0.25	-0.36	-0.30	-0.40	-0.11	-0.22	-0.27	-0.30

Prior to EFA, the Solomon method was used to assess the equivalence of the subsamples in each school, and the community ratio index (CRI) was calculated as a measure for the similarities between the subsamples, with a high value (close to unity) indicating a high degree of equivalence. The CRI values for the middle school and elementary school samples were close to unity (CRI: elementary school = .987; middle school = .961), which suggested that these subsamples were representative of the same populations.

In addition, the Kaiser–Meyer–Olkin (KMO) index was used to ensure the data appropriateness for the EFA on each subsample. All subsamples had values more than 0.6, which indicated that the data were suitable for performing the EFA (minimum KMO: elementary school = .763; middle school = .762).

The parallel analysis found that the two-factor model was the most appropriate for the elementary and middle school samples, which explains 43.2% and 41.7% of the total variance, respectively. Table 2 shows the standardized factor loadings and eigenvalues obtained from two-factor model. All individual items were loaded onto their corresponding constructs with loading values greater than .35. The estimated correlations between the two factors for the elementary and middle school samples were .47 and .40, respectively.

**Table 2:** Standardized factor loadings for two-factor model of the Grit-S at baseline.

Item	Elementary		Middle	
	Factor 1	Factor 2	Factor 1	Factor 2
**1. New ideas and projects sometimes distract me from previous ones**	.714	.050	.682	.020
**3. I have been obsessed with a certain idea or project for a short time, but later lost interest.**	.773	-.012	.739	-.008
**5. I often set a goal but later choose to pursue a different one.**	.506	-.172	.599	-.239
**6. I have difficulty maintaining my focus on projects that take more than a few months to complete.**	.611	.180	.674	.053
**2. Setbacks do not discourage me.**	-.063	.486	-.065	.397
**4. I am a hard worker.**	.010	.725	-.002	.770
**7. I finish whatever I begin.**	-.030	.665	.177*	.573
**8. I am diligent.**	.045	.669	.132*	.539

**Fig 1:**
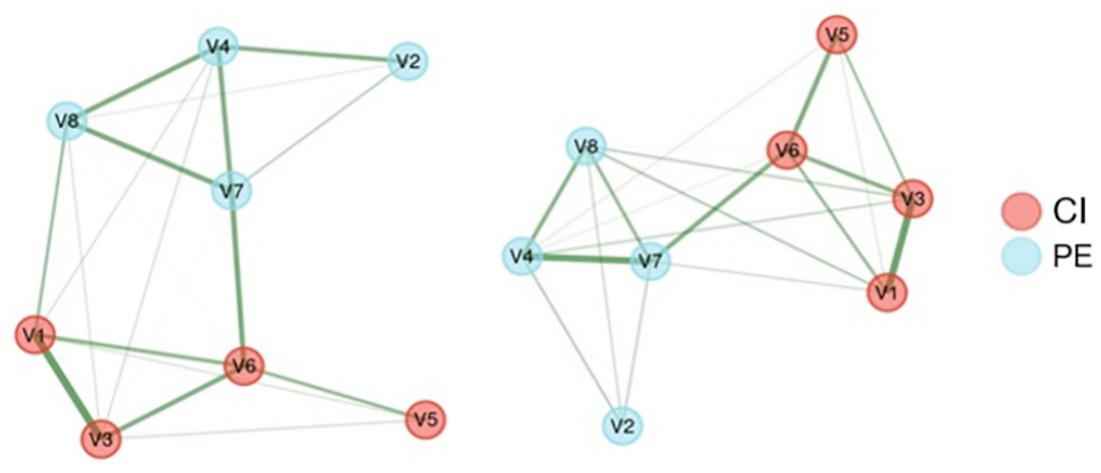
Estimated network models for the Grit-S at the first time in the elementary and middle school samples. The panel on the left-hand side represents an elementary school’s network, and the panel on right-hand side represents a middle school’s network. In the estimated network, CI indicates consistency of interest factor and PE shows perseverance of the effect factor.

Fig 1 shows the estimated Grit-S network. For both samples, two item dimensions were identified using *bootEGA*, which was the same solution as the EFA. The results of bootstrap iterations allowed for an examination of the dimensional stability found that the two-dimension solution exhibited a 99.5% replication rate (995 out of 1,000 samples) in the elementary school sample and a 100.0% replication rate (1,000 out of 1,000 samples) in the middle school sample. All items loaded perfectly on their respective dimensions in both samples.

The CFA revealed that the two-factor model selected using EFA and EGA was superior to the one‐factor model for both samples (elementary school: χ^2^ = 103.5, *df* = 19, CFI = .975, SRMR = .050, RMSEA = .062; middle school: χ^2^ = 121.3, *df* = 19, CFI = .968, SRMR = .054, RMSEA = .068), as shown in Table 3. Table 3 also reveals that the CFI value obtained from the two-factor model exceeded the strictest DFI cutoff levels (Level-1) in the elementary school sample even though the empirical values for SRMR and RMSEA were slightly higher than the cutoff values.

**Table 3:** Fit indices for the measurement models.

School	Models	Model fit index					^a^DFI cutoffs		
		χ^2^	*df*	^b^CFI	^c^SRMR	^d^RMSEA [90% CI]	^b^CFI	^c^SRMR	^d^RMSEA
**Elementary**	**One-factor** ^e^	412.7	20	.884	.099	.130 [.119, .141]	.966	.033	.047
	**Two factors**	103.5	19	.975	.050	.062 [.051, 074]	.974	.029	.046
**Middle**	**One-factor** ^e^	463.1	20	.862	.105	.138 [.127, .149]	.957	.034	.051
	**Two factors**	121.3	19	.968	.054	.068 [.057, .080]	.980	.027	.039

^a^ DFI cutoffs = Dynamic fit index cutoff;

^b^ CFI = comparative fit index;

^c^ SRMR = standardized root mean square residual;

^d^ RMSEA = root mean square error of estimators;

^e^ All indicators load on a single factor.

All empirical fit indices deteriorated in comparison to the DFI cutoff in the middle school sample. Nevertheless, the two-factor model exhibited a relatively smaller discrepancy between the empirical fit measures and the dynamic fit measure cutoffs compared to the one-factor model. Based on these findings, the two-factor model was selected as the final model to adequately explain the Grit-S factor structure for both samples, and the following analyses were conducted using the two-factor model. S2 and S3 Tables present the CFA standardized factor loadings obtained for both samples.

### Cross-Sectional measurement invariance

Table 4 displays the results of the CMI analyses across genders. The configural model showed an adequate fit to the data for both samples (elementary school: χ^2^ = 483.670, *df* = 38, CFI = .947, SRMR = .051, RMSEA = .100; middle school: χ^2^ = 564.517, *df* = 38, CFI = .927, SRMR = .056, RMSEA = .109). Subsequently, we established a thresholds invariance (TI) model by restricting the item thresholds to be equal across genders. The fit of the TI model did not deteriorate more than the cutoff values from the fit obtained using the configural model in the elementary school sample (Δχ^2^ = 7.370, *df* = 8, *p* = .050; RMSEA_D_. = 031), but the difference in the middle school sample was significant (Δχ^2^ = 29.957, *df* = 8, *p* < .001; RMSEA_D_. = 045). These results indicated that the threshold invariance across genders was supported only in the elementary school sample; however, we found a lack of full invariance for the thresholds in the middle school sample.

**Table 4:** Measurement invariance for the Grit-S across gender at baseline.

School	Invariance model	Model fit index					Measurement invariance test			
		χ^2^	df	CFI	SRMR	RMSEA	Δ χ^2^	Δdf	*p*	RMSEA_D_
**Elementary** **(N = 2,327)**	^a^ **Configural**	483.670	38	.947	.051	.100 [.093, .109]				
	^b^ **TI**	494.842	46	.946	.051	.092 [.084, .099]	7.370	8	.050	.031 [.022,.041]
	^c^ **T&LI**	430.923	52	.955	.052	.079 [.072, .086]	6.878	6	.332	.042 [.033,.052]
**Middle** **(N = 2,325)**	^a^ **Configural**	564.517	38	.927	.056	.109 [.101, .117]				
	^b^ **TI**	592.890	46	.924	.056	.101 [.094, .109]	29.597	8	< .001	.045 [.011,.039]
	^d^ **PTI**	570.521	44	.927	.056	.102 [.094, .109]	6.5003	6	.370	.022 [.012,.033]
	^c^ **T&LI**	542.199	50	.931	.059	.092 [.085, .099]	39.561	6	< .001	.057 [.045,.073]
	^e^ **PT&LI**	469.816	48	.941	.057	.087 [.080, .094]	14.561	4	.057	.044 [.028,.063]

^a^ Configural = configural model;

^b^ TI = threshold invariance model;

^c^ T&LI = threshold and loading invariance model;

^d^ PTI = partial threshold invariance model;

^e^ PT&LI = partial threshold and loading invariance

Several studies have found that as partial invariance is sufficient for achieving MI if the proportion of the noninvariant items is small in a scale [63, 64], it can be accommodated to proceed the subsequent group comparisons [65–67]. The partial threshold invariance (PTI) model was formed by relaxing the equality constraint on the second thresholds for items 7 and 8, which exhibited the two largest modification index values. Table 4 demonstrates that the difference in fit measures between the configural and PTI model was nonsignificant (Δχ^2^ = 6.5003, *df* = 6, *p* = .370; RMSEA_D_. = 022), that is, the PTI was supported for the middle school sample.

The threshold and loading invariance (T&LI) models were established by imposing constraints on both thresholds and factor loadings to be equal across genders. The difference in the fit index between the threshold invariance model (full and partial) and T&LI models was significant for the middle school sample (Δχ^2^ = 39.561, *df* = 6, *p* < .001; RMSEA_D_ = .057). However, the difference between the two models was nonsignificant for the elementary school sample (Δχ^2^ = 6.878, *df* = 6, *p* = .332; RMSEA_D_ = .032). These results indicated that the full threshold and loading invariance across genders was supported only in the elementary school sample, but was not maintained in the middle school sample.

Therefore, the partial thresholds and loading invariance (PT&LI) model was built by relaxing the equality constraint of factor loadings on items 1 (*New ideas and projects sometimes distract me from previous ones*) and 5 (*I often set a goal but later choose to pursue a different one*), both of which had the two largest loading differences between genders in the configural model. Table 4 reveals that the difference in fit measures between the PTI and PT&LI models was nonsignificant (Δχ^2^ = 14.561, *df* = 4, *p* = .057; RMSEA_D_ = .044), which indicates that partial thresholds and loading invariance was supported for the middle school sample.

### Longitudinal measurement invariance

Table 5 lists the results of the LMI. First, model fit was separately assessed at each time point. For both samples, all models displayed a satisfactory fit with the CFI, SRMR, and RMSEA values, which enabled to perform a further test of the LMI.

**Table 5:** Longitudinal measurement invariance for the Grit-S over time.

School	Invariance model	Model fit index					Measurement invariance test			
		χ^2^	df	CFI	SRMR	RMSEA	Δ χ^2^	Δdf	*p*	RMSEA_D_
**Elementary** **(N = 2,327)**	**Time 1**	177.903	19	.940	.043	.060 [.052, .068]				
	**Time 2**	174.202	19	.931	.044	.059 [.051, .067]				
	**Time 3**	145.034	19	.943	.039	.053 [.045, .062]				
	^a^ **Configural**	942.565	213	.953	.034	.038 [.036, .041]				
	^b^ **TI**	951.213	229	.954	.034	.037 [.034, .039]	37.667	16	.002	.031 [.022,.041]
	^c^ **PTI**	936.337	226	.955	.034	.037 [.034, .039]	21.149	13	.070	.023 [.012,.034]
	^d^ **T&LI**	885.602	238	.959	.035	.034 [.032, .037]	26.220	12	.009	.038 [.029,.050]
	^e^ **PT&LI**	878.307	237	.959	.035	.034 [.032, .036]	15.478	11	.161	.027 [.016,.040]
**Middle** **(N = 2,325)**	**Time 1**	192.302	19	.922	.045	.060 [.052, .068]				
	**Time 2**	110.006	19	.951	.034	.059 [.051, .067]				
	**Time 3**	214.232	19	.910	.049	.053 [.045, .062]				
	^a^ **Configural**	1118.412	213	.945	.036	.043 [.040, .045]				
	^b^ **TI**	1099.299	229	.947	.036	.040 [.038, .043]	29.328	16	.022	.025 [.016,.035]
	^c^ **PTI**	1096.557	228	.947	.036	.040 [.038, .043]	24.138	15	.063	.022 [.012,.033]
	^d^ **T&LI**	1002.897	240	.953	.037	.037 [.035, .040]	29.472	12	< .001	.046 [.036,.056]
	^e^ **PT&LI**	1007.163	239	.953	.037	.037 [.035, .040]	17.359	11	.098	.032 [.021,.044]

^a^ Configural = configural model;

^b^ TI = threshold invariance model;

^c^ PTI = partial threshold invariance model;

^d^ T&LI = threshold and loading invariance model;

^e^ PT&LI = partial threshold and loading invariance

Table 5 also indicates that the differences between the configural and TI models were statistically significant for both samples (elementary school: Δχ^2^ = 37.667, *df* = 16, *p* = .002; RMSEA_D_ = .031; middle school: Δχ^2^ = 29.328, *df* = 16, *p* = .022; RMSEA_D_ = .025), which suggested that the full threshold invariance was not supported for both samples. The PTI model was constructed by releasing the equality constraint over time for the second thresholds of item 1 at time 2 and 3 and item 4 at time 3 in the elementary school sample and the second thresholds of item 7 in the middle school sample. No significant differences were found between the configural and PTI models for both samples (elementary school: Δχ^2^ = 21.149, *df* = 13, *p* = .070; RMSEA_D_ = .023; middle school: Δχ^2^ = 24.138, *df* = 15, *p* = .063; RMSEA_D_ = .022), which indicated that the threshold invariance was partially supported for both samples.

Table 5 also shows that the differences in the fit index between the PTI and T&LI models were significant for both school samples (elementary school: Δχ^2^ = 26.220, *df* = 12, *p* = .009; RMSEA_D_ = .038; middle school: Δχ^2^ = 29.472, *df* = 12, *p* < .001; RMSEA_D_ = .046). This finding implied that the factor loadings did not remain invariant over time for both samples. Therefore, the PT&LI models were constructed by freely estimating the factor loadings for item 3 at time 2 in the elementary school sample and for item 5 at time 2 in the middle school sample.

It was found that the differences in the fit index between the partial metric and partial scalar models were nonsignificant for both samples (elementary school: Δχ^2^ = 15.478, *df* = 11, *p* = .161; RMSEA_D_ = .027; middle school: Δχ^2^ = 17.359, *df* = 11, *p* = .098; RMSEA_D_ = .032), which indicated that the partial T&LI was supported for both samples.

In summary, these findings suggested that the Grit-S exhibited LMI over time for the configuration, and partial threshold and loading in both school samples. S4 and S5 Tables show the standardized factor loadings for the longitudinal invariance model.

### Reliability analyses

The internal consistency evaluated using McDonald’s ω was acceptable (ω > 0.70) at all time points, with the ω coefficients respectively being .716, .700, and .712 at times 1, 2, and 3 in the elementary school sample, and .716, .700, and .712 at the same time points in the middle school sample.

The partial T&LI models were employed to examine the stability of the factors over time. The estimated factor correlations between times 1 and 2 were .403 and .474 for CI and PE in the elementary school sample and .519 and .562 in the middle school sample, all of which were statistically significant (*p* < .001). These findings suggested that the internal consistencies of the three factors were generally acceptable and that the factors demonstrated moderate stability over time.

## Discussion

The major findings of this study were as follows. First, the CFA results indicated that the Korean version of the Grit-S had a two-factor structure in both the elementary and middle school samples. Second, the CMI analyses across genders indicated that the full and partial thresholds and the loading invariance were supported in both the elementary and middle school samples. Third, the LMI results revealed that the scale had partial T&LI in both samples. Finally, the scale reliability as measured by McDonald’s ω was acceptable, and the factors had moderate stability over time.

This study provides a more comprehensive understanding of the applicability of the Grit-S. First, the Grit-S was found to have a two-factor structure in both the Korean elementary and middle school samples. Because of the diverse Grit-S factor structures in previous studies, it was suggested that the Grit-S factor structure needed to be assessed in different cultures and age groups [27, 31]. Therefore, this study addressed these research gaps for early adolescent samples.

The results of this study were inconsistent with the Grit-S EFA results (i.e., the unidimensional model) from a study on early adolescents in the U.S. [68]. However, it has been suggested that the grit factor structure could differ across cultures due to the diverse values and beliefs in specific cultural groups [28, 29]. For example, individualist cultures highlight self-set goals whereas collectivist cultures tend to prioritize interpersonal harmony and the pursuit of group goals over individual goals [69, 70]. Due to a tendency to set goals that align with and adapt to significant others, students in collectivistic cultures may place less emphasis on consistency of interest than on persistence of effort in the two grit components [28], that is, people from collectivistic cultures may perceive grit as comprising two distinct but correlated constructs rather than seeing it as a combination of both CI and PE. Although further research is necessary, this study’s findings contribute to the ongoing discussion regarding cross-cultural differences in the grit structure.

Second, studies have agreed that a meaningful comparison of factors or observed means is possible when the scalar invariance level (factor loadings and intercepts) is supported [65, 66, 71, 72]. This verified that the Korean version of the scale had approximate scalar cross-sectional and longitudinal invariance in both school samples. Some studies have suggested that a meaningful comparison of the scale factor means is possible when partial invariance is supported [66, 71, 72] when the proportion of noninvariant items in the scale is small [63, 64]. As this study found sufficient evidence of configural, threshold, and loading invariance in the two MI aspects that corresponds to the scalar invariance, it was concluded that the Korean version of the Grit-S satisfied the CMI and the LMI in both samples. Therefore, as the study’s findings aligned with the reliability analyses, the Korean version of the Grit-S was deemed to have satisfactory psychometric properties for Korean adolescents. These results also indicated that the observed gender differences in the scale and the changes in the scale over time could be interpreted as actual differences and changes in the measured construct.

This study also highlighted relevant practical implications for future research. First, as the sample data size was sufficiently large and the data were collected using probability sampling, generalizability is possible to the Korean adolescent population. Second, the majority of previous studies tended to focus on separately reporting the CMI or LMI. To the best of our knowledge, this study is the first to simultaneously examine the CMI and LMI of the Grit-S based on an estimator that analyzed the ordered categorical data, which is an important contribution because it confirmed that the gender differences in the obtained scores reflect true gender differences and the increases in the observed scores over time were actual developmental changes in the given grit construct.

Regardless of the novel findings, this study had a few limitations. First, the samples were collected only from elementary and middle school settings. Therefore, future studies should examine adult samples to enhance the generalizability of the findings. Second, the psychometric properties of the scale were only investigated in a collectivist culture. Future studies should, therefore, seek to identify the cross-cultural differences in other collectivist and individualist societies. Third, attrition is inevitable in longitudinal research, and this study was not exempt. In the baseline model, 2,607 elementary students responded; however, this decreased to 2,327 in the third year (a dropout rate of approximately 10.7%). At baseline, 2,590 middle school students participated; however, this decreased to 2,325 in the third year (a dropout rate of approximately 10.2%). Whether the participants who remained differed significantly from those who dropped out is unknown. As missing cases can lead to problems in longitudinal studies, additional analyses were conducted to examine the impacts of the missing data, which were input and then used to create complete datasets using the expectation-maximization algorithm. The results from these newly created datasets were compared with those of the main study and were found to be similar, which implied that the missing data had not had any serious influence on the overall conclusions.

## Supporting information

S1 Table Demographic characteristics (elementary: N = 2,327; middle: N = 2,325). (DOCX)

S2 Table Standardized factor loadings for two-factor model of the Grit-S in elementary school at baseline. (DOCX)

S3 Table Standardized factor loadings for the two-factor model of the Grit-S in middle school at baseline. (DOCX)

S4 Table Standardized factor loadings in the configural invariance model of the Grit-S. (DOCX)

S5 Table Standardized factor loadings in the configural invariance model of the Grit-S. (DOCX)
