# Supplementary material for: Psychometric properties and measurement invariance of the short form of grit scale in Korean adolescents
Source: PLoS One. 2024 Jan 19;19(1):e0296795. doi: 10.1371/journal.pone.0296795 (PMC10798495; doi:10.1371/journal.pone.0296795)
Supplement: S1 Table — (DOCX) [file pone.0296795.s001.docx]

**S1 Table. Demographic Characteristics (Elementary: N = 2,327; Middle: N = 2,325)**

| Variable | Category | Elementary  n (%) | Middle  n (%) | χ^2^/*p*-value |
| --- | --- | --- | --- | --- |
| Gender | Male | 1,168 (50.2) | 1,248 (53.7) | 5.655/.017 |
|  | Female | 1,159 (49.8) | 1,077 (46.3) |  |
| Urban scale | Metropolitan city | 956 (41.1) | 980 (42.2) | .835/.659 |
|  | Medium-sized city | 1,010 (43.4) | 1,003 (43.1) |  |
|  | Rural town/village | 361 (15.5) | 342 (14.7) |  |
| Economic level perceived subjectively | Lowest | 35 (1.5) | 28 (1.2) | 5.448/.244 |
|  | Low | 270 (11.6) | 283 (12.2) |  |
|  | Average | 1,813 (77.9) | 1,773 (76.3) |  |
|  | High | 193 (8.3) | 225 (9.7) |  |
|  | Highest | 6 (0.3) | 11 (0.5) |  |
|  | No response | 10 (0.4) | 5 (0.2) |  |
