# Supplementary material for: Psychometric properties and measurement invariance of the short form of grit scale in Korean adolescents
Source: PLoS One. 2024 Jan 19;19(1):e0296795. doi: 10.1371/journal.pone.0296795 (PMC10798495; doi:10.1371/journal.pone.0296795)
Supplement: S2 Table — (DOCX) [file pone.0296795.s002.docx]

**S2 Table. Standardized Factor Loadings for Two-Factor Model of the Grit-S in Elementary School at Baseline**

| Item | Loadings | *SE* | *p-Value* | 95% CI |
| --- | --- | --- | --- | --- |
| Consistency of interest |  |  |  |  |
| 1. New ideas and projects sometimes distract me from previous ones | .736 | .021 | .000 | [.695, .777] |
| 3. I have been obsessed with a certain idea or project for a short time, but later lost interest. | .718 | .022 | .000 | [.676, .761] |
| 5. I often set a goal but later choose to pursue a different one. | .328 | .028 | .000 | [.273, .383] |
| 6. I have difficulty maintaining my focus on projects that take more than a few months to complete. | .736 | .022 | .000 | [.692, .779] |
| Perseverance of effort |  |  |  |  |
| 2. Setbacks do not discourage me. | .387 | .027 | .000 | [.334, .439] |
| 4. I am a hard worker. | .710 | .022 | .000 | [.667, .753] |
| 7. I finish whatever I begin. | .713 | .022 | .000 | [.669, .756] |
| 8. I am diligent. | .730 | .019 | .000 | [.692, .768] |
