# Supplementary material for: Psychometric properties and measurement invariance of the short form of grit scale in Korean adolescents
Source: PLoS One. 2024 Jan 19;19(1):e0296795. doi: 10.1371/journal.pone.0296795 (PMC10798495; doi:10.1371/journal.pone.0296795)
Supplement: S3 Table — (DOCX) [file pone.0296795.s003.docx]

**S3 Table. Standardized Factor Loadings for the Two-Factor Model of the Grit-S in Middle School at Baseline**

| Item | Loadings | *SE* | *p-Value* | 95% CI |
| --- | --- | --- | --- | --- |
| Consistency of interest |  |  |  |  |
| 1. New ideas and projects sometimes distract me from previous ones | .739 | .020 | .000 | [.701, .778] |
| 3. I have been obsessed with a certain idea or project for a short time, but later lost interest. | .742 | .021 | .000 | [.701, .782] |
| 5. I often set a goal but later choose to pursue a different one. | .397 | .028 | .000 | [.344, .451] |
| 6. I have difficulty maintaining my focus on projects that take more than a few months to complete. | .739 | .020 | .000 | [.700, .778] |
| Perseverance of effort |  |  |  |  |
| 2. Setbacks do not discourage me. | .271 | .031 | .000 | [.211, .331] |
| 4. I am a hard worker. | .638 | .023 | .000 | [.592, .684] |
| 7. I finish whatever I begin. | .731 | .023 | .000 | [.686, .775] |
| 8. I am diligent. | .744 | .022 | .000 | [.700, .788] |
