# Supplementary material for: Psychometric properties and measurement invariance of the short form of grit scale in Korean adolescents
Source: PLoS One. 2024 Jan 19;19(1):e0296795. doi: 10.1371/journal.pone.0296795 (PMC10798495; doi:10.1371/journal.pone.0296795)
Supplement: S5 Table — (DOCX) [file pone.0296795.s005.docx]

**S5 Table. Standardized Factor Loadings in the Configural Invariance Model of the Grit-S**

| Item | Elementary | | | | Middle | | |
| --- | --- | --- | --- | --- | --- | --- | --- |
|  | Time 1 | Time 2 | Time 3 | Time 1 | | Time 2 | Time 3 |
| Consistency of interest |  |  |  |  | |  |  |
| 1. New ideas and projects sometimes distract me from previous ones. | .695 | .613 | .607 | .536 | | .501 | .484 |
| 1. I have been obsessed with a certain idea or project for a short time, but later lost interest. | .676 | .654 | .636 | .533 | | .492 | .525 |
| 1. I often set a goal but later choose to pursue a different one. | .540 | .512 | .530 | .258 | | .310 | .310 |
| 1. I have difficulty maintaining my focus on projects that take more than a few months to complete. | .697 | .619 | .662 | .497 | | .458 | .515 |
| Perseverance of effort |  |  |  |  | |  |  |
| 1. Setbacks do not discourage me. | .578 | .500 | .507 | .196 | | .189 | .181 |
| 1. I am a hard worker. | .605 | .589 | .548 | .451 | | .415 | .409 |
| 1. I finish whatever I begin. | .661 | .632 | .576 | .491 | | .460 | .476 |
| 1. I am diligent. | .587 | .564 | .568 | .528 | | .464 | .524 |
